# Potential novel biomarkers for chronic lung allograft dysfunction and azithromycin responsive allograft dysfunction

**DOI:** 10.1038/s41598-021-85949-1

**Published:** 2021-03-24

**Authors:** Cecilia Veraar, Jonathan Kliman, Alberto Benazzo, Felicitas Oberndorfer, Maria Laggner, Philipp Hacker, Thomas Raunegger, Stefan Janik, Peter Jaksch, Walter Klepetko, Hendrik J. Ankersmit, Bernhard Moser

**Affiliations:** 1 Division of Cardiac Thoracic Vascular Anaesthesia and Intensive Care Medicine, Department of Anaesthesiology, General Intensive Care and Pain Medicine, Medical University of Vienna, Vienna, Austria; 2 Division of Surgery, Department of Thoracic Surgery, Medical University of Vienna, Vienna, Austria; 3 Clinical Institute of Pathology, Medical University of Vienna, Vienna, Austria; 4 Department of Otorhinolaryngology, Head and Neck Surgery, Medical University of Vienna, Vienna, Austria; 5 Christian Doppler Laboratory for Diagnosis and Regeneration of Cardiac and Thoracic Diseases, Medical University of Vienna, Vienna, Austria; 6 Head FFG Project “APOSEC”, FOLAB Surgery, Medical University of Vienna, Vienna, Austria; 7 Division of Thoracic Surgery, Department of Surgery, Medical University of Vienna, Waehringer Guertel 18-20, 1090 Vienna, Austria

**Keywords:** Cytokines, Diagnosis, Translational research

## Abstract

Chronic Lung Allograft Dysfunction (CLAD), manifesting as Bronchiolitis Obliterans Syndrome (BOS) or Restrictive Allograft Syndrome (RAS), is the main reason for adverse long-term outcome after Lung Transplantation (LTX). Until now, no specific biomarkers exist to differentiate between CLAD phenotypes. Therefore, we sought to find suitable cytokines to distinguish between BOS, RAS and Azithromycin Responsive Allograft Dysfunction (ARAD); and reveal potential similarities or differences to end-stage fibrotic diseases. We observed significantly increased Lipocalin-2 serum concentrations in RAS compared to BOS patients. In addition, in RAS patients immunohistochemistry revealed Lipocalin-2 expression in bronchial epithelium and alveolar walls. Patients with ARAD showed significantly lower Activin-A serum concentrations compared to Stable-LTX and BOS patients. Further, increased serum concentrations of Lipocalin-2 and Activin-A were predictors of worse freedom-from-CLAD in Stable-LTX patients. These biomarkers serve as promising serum biomarkers for CLAD prediction and seem suitable for implementation in clinical practice.

## Introduction

Lung Transplantation (LTX) is performed as the last therapeutic option for end-stage pulmonary fibrotic diseases, including Idiopathic Pulmonary Fibrosis (IPF) and Cystic Fibrosis (CF). Five- and ten-year survival rates of adults after primary LTX are 55–60% and 34%, respectively. Despite gradually improving perioperative survival rates, long-term outcomes remained almost unaffected. Chronic Lung Allograft Dysfunction (CLAD) remains the main limiting factor for reduced life expectancy after LTX occurring in approximately 50% of transplant recipients within 5 years after primary LTX^1,2^.

There are two predominant CLAD phenotypes: bronchiolitis obliterans syndrome (BOS) and Restrictive Allograft Syndrome (RAS)^3^. Bronchiolitis Obliterans (BO) after LTX manifests as narrowing or complete occlusion of the small airways. As the histopathological diagnosis of BO is hard to obtain, BOS was introduced as the clinical correlate of BO, characterized by an irreversible airway obstruction^2^. RAS, contributing to 25–35% of CLAD has an even worse prognosis than BOS with an average life expectancy of 540 versus 1200 days. RAS is histologically characterised by diffuse alveolar damage and extensive fibrosis of the alveolar interstitium^4,5^. Before CLAD can be diagnosed, allograft-related (e.g. persistent acute rejection, Azithromycin Responsive Allograft Dysfunction (ARAD), infection/colonization, anastomotic stricture) and extra-allograft-related (e.g. pleural disease, diaphragmatic dysfunction) confounding factors leading to non-chronic rejection with Forced Expiratory Volume in one second (FEV_1_) decline have to be excluded^6^.

IPF is the most lethal fibrotic disease of the lung. Healthy tissue is replaced by altered extracellular matrix deposition and alveolar architecture is destroyed, leading to decreased lung compliance, disrupted gas exchange, and ultimately respiratory failure and death^7^. CF is a genetic multi-systemic disorder affecting lungs, pancreas, liver and intestine^8^. Mutations of the Cystic Fibrosis Transmembrane Conductance Regulator (CFTR) gene cause impaired mucociliary clearance leading to mucus plugging, opportunistic infections, chronic inflammation, oxidative stress and airway remodeling^9^. Although the etiologies of IPF, CF and CLAD significantly differ, the pathophysiology of these diseases show overlapping characteristics, including epithelial cell injury, increased production and deposition of extracellular matrix, immune cell activation, and fibroblast proliferation^10^.

In human lungs Lipocalin-2 is expressed in small quantities under healthy conditions and is up-regulated during pulmonary disease, infection and lung injury^11^. In CF patients Lipocalin-2 was increased in bronchial secretions and thereby reflecting neutrophilic inflammation^12^. In low concentrations Lipocalin-2 is involved in innate immunity by acting as a siderophore, causing iron depletion to prevent bacterial growth and exhibits anti-inflammatory effects on macrophages^13^.

The imbalance between Matrix Metalloproteinases (MMPs) and Tissue Inhibitors of Metalloproteinases (TIMPs) might be an important trigger for fibrogenic processes. Elevated MMP-9 concentrations and MMP-9/TIMP-1 ratios in serum and Bronchioalveolar Lavage (BAL) were associated with CLAD, IPF and CF^14^.

Activin-A belongs to the transforming growth factor-β superfamily and is produced by immune, epithelial and endothelial cells. Activin-A promotes fibrotic processes by stimulating fibroblast proliferation, differentiation and inducing regulators of fibrosis^15^. Follistatin is an activin-binding protein that inhibits the activity of Activin-A. Thereby, Follistatin is capable of reducing inflammation, fibrosis and mortality^16^.

So far the diagnosis of CLAD remains limited to clinical parameters reflecting permanent loss of lung function. We aimed to identify novel biomarkers for early CLAD diagnosis, prior to emerging irreversible lung injury and refine distinguishing biomarkers for ARAD and CLAD phenotypes: BOS and RAS, associated with different prognosis and thereby enabling early interventions that could modify or even avoid the inevitable degradation of pulmonary function.

The aim of the study was to shed light on the cytokine pathophysiology of patients with fibrotic diseases, including LTX patients with CLAD (BOS and RAS) and ARAD; and patients with end-stage pulmonary fibrosis (IPF and CF).

The loss of pulmonary function in a patient after LTX will prompt a series of diagnostic investigations. The first clinical question that arises is about reversibility of the observed pulmonary function loss. After a thorough workup excluding extra-CLAD causes of pulmonary function loss (e.g. silent aspiration), patients not responding to azithromycin are only identified after 3 months of unsuccessful azithromycin treatment. Thus the first objective of this study was to identify biomarkers able to distinguish patients with ARAD from those with CLAD and to investigate whether treatment responses to azithromycin are predictable via cytokines. After ARAD is excluded clinicians want to distinguish the different forms of CLAD: BOS and RAS. So, our second objective was to identify biomarkers that can help to distinguish BOS from RAS. We believe that different biomarkers could be a helpful additive diagnostic tool in decision-making at certain crossroads (ARAD vs. CLAD or BOS vs. RAS) in the context of clinical workup and follow-up. Apart from these clinical decision-making algorithms in LTX patients our third objective was of a purely academic interest contributing to better understanding of possible similarities or differences between the pathophysiology of CLAD and end-stage fibrotic diseases such as CF and IPF. Regarding prognosis, we sought to determine biomarkers capable of predicting freedom-from-CLAD. We therefore sought to investigate the outcome of CLAD in the context of biomarkers (outcome: overall survival (OS)/re-LTX).

## Results

Cytokine serum concentrations of patients with Stable-LTX, ARAD, CLAD (BOS and RAS), end-stage IPF and CF as well as healthy volunteers were summarized in Table 1. Intra-assay and inter-assay Coefficients of Variability (CV) can be found in the Supplementary Table 1. The lowest intra-assay %CV was at 1.0 for Lipocalin-2, the highest %CV was at 3.4 for Folliastatin. The lowest inter-assay %CV was calculated for MMP-9, while the highest inter-assay %CV was measured for TIMP-1.

**Table 1:** Comparison of cytokine serum concentrations.

	LCN-2 (ng/ml)	MMP-9 (ng/ml)	TIMP-1 (ng/ml)	MMP-9/TIMP-1 ratio	Activin-A (pg/ml)	FST (pg/ml)	Activin-A/FST ratio
Healthy (n = 63)	344.6 [126.2–389.3]	559.9 [304.0–1013.0]	44.4 [33.6–106.8]	11.2 [4.9–28.2]	362.8 [304.6–544.5]	207.8 [141.3–438.2]	1.6 [0.7–3.3]
Stable-LTX (n = 63)	365.7 [313.5–510.9]	511.0 [321.7–1246.4]	134.4 [60.1–319.2]	4.5 [1.8–10.6]	369.0 [196.9–505.5]	333.1 [129.5–505.5]	1.1 [0.4–3.5]
ARAD (n = 22)	368.4 [276.8–485.5]	490.4 [315.9–1314.8]	93.0 [40.6–213.9]	6.8 [1.9–17.3]	118.1 [72.6–343.0]	363.8 [180.1–721.9]	0.4 [0.1–0.9]
CLAD (n = 41)	388.6 [310.4–602.8]	908.0 [467.4–1552.8]	105.9 [57.6–319.7]	7.5 [2.5–17.7]	371.1 [205.1–994.5]	297.1 [232.6–942.3]	1.1 [0.5–2.7]
BOS (n = 30)	367.0 [284.1–584.2]	879.4 [537.7–1619.9]	93.7 [49.6–209.8]	8.1 [2.8–22.6]	343.9 [212.3–759.1]	268.0 [182.0–1023.7]	1.0 [0.6–2.3]
RAS (n = 11)	518.4 [444.8–768.2]	1073.2 [289.6–1546.9]	199.4 [103.3–486.9]	2.7 [2.1–9.6]	883.8 [84.7–2295.4]	387.8 [295.4–903.0]	1.7 [0.3–6.6]
IPF (n = 23)	321.6 [162.0–379.2]	1634.7 [1115.2–2429.4]	121.0 [50.9–434.0]	11.8 [3.0–42.3]	327 [212.8–636.4]	210.5 [177.7–257.7]	1.4 [0.7–3.6]
CF (n = 21)	366.3 [282.3–592.8]	2186.7 [1526.9–3791.5]	220.8 [62.6–505.1]	8.6 [3.8–39.0]	377 [212.8–636.4]	207.6 [153.8–308.2]	1.7 [0.8–3.8]

Serum cytokine concentrations of healthy volunteers, Stable-LTX, ARAD, CLAD (RAS and BOS) and patients with end-stage pulmonary fibrosis (IPF and CF) were measured by immunoassays and reported as median [25–75% Percentile].

*n* number of individuals, *LCN-2* Lipocalin-2, *MMP-9* Matrix Metalloproteinase-9, *TIMP-1* Tissue Inhibitor of Matrix Metalloproteinase, *FST* Follistatin, *Stable-LTX* transplanted patients free from CLAD, *ARAD* azithromycin-responsive allograft dysfunction, *CLAD* chronic lung allograft dysfunction, *BOS* bronchiolitis obliterans syndrome.

*RAS* restrictive allograft syndrome, *IPF* idiopathic pulmonary fibrosis, *CF* cystic fibrosis.

### Lipocalin-2 serum concentrations differentiate RAS from BOS, ARAD, and Stable-LTX

**Figure 1:**
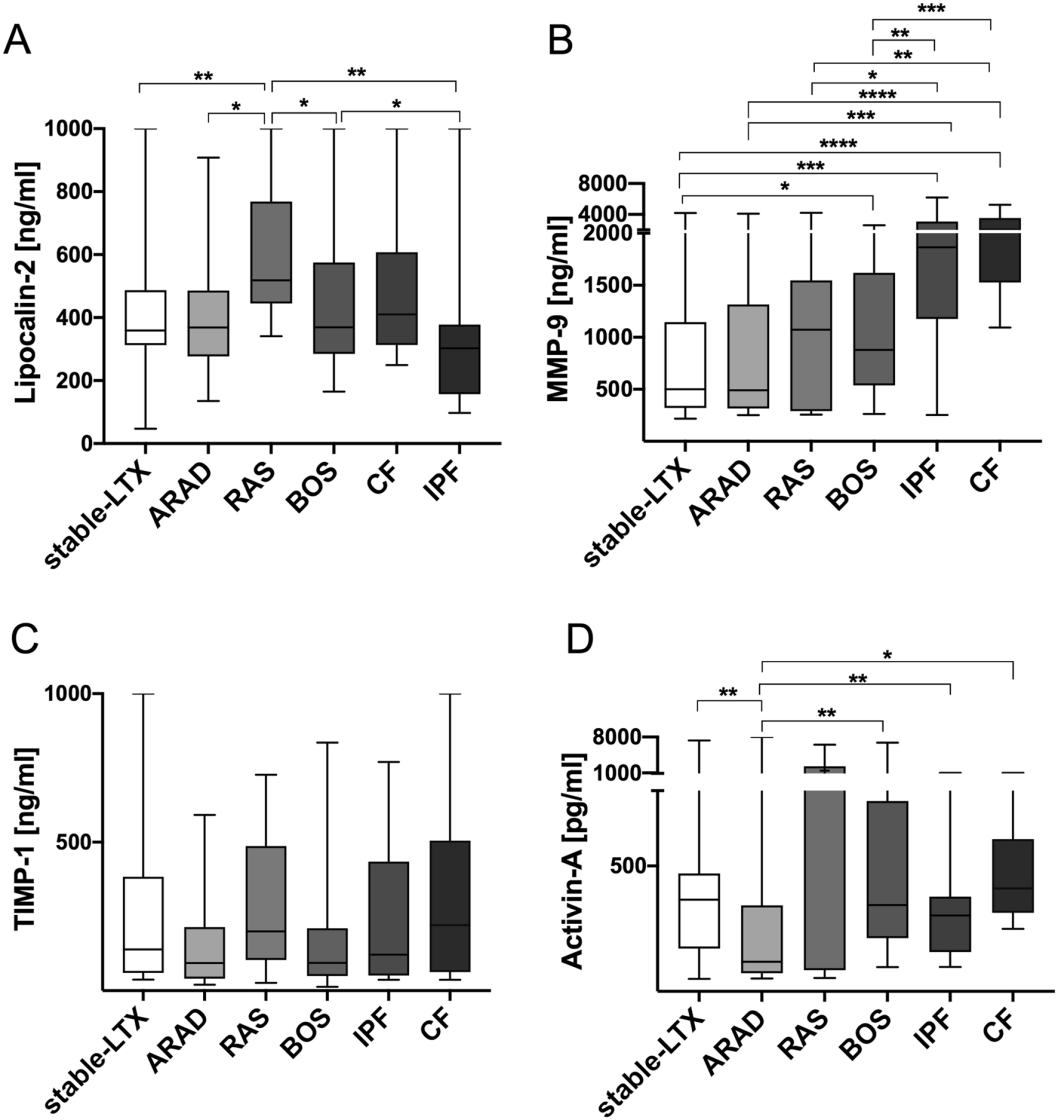
Differential expression of Lipocalin-2, Activin-A and MMP-9. Statistically significant differences for Lipcalin-2, MMP-9 and Activin-A are depicted in (**A**,**B**) and (**D**) where applicable. There were no statistically significant differences for TIMP-1 serum concentrations between all groups (**C**). Mann–Whitney U testing was performed between two groups each. The graphical depiction of all six groups together was chosen for better overview. The following p-values ware not corrected for multiple testing. *p < 0.05; **p < 0.01; ***p < 0.001 *RAS* Restrictive allograft syndrome, *BOS* bronchiolitis obliterans syndrome, *ARAD* azithromycin-responsive allograft dysfunction, *MMP-9* matrix metalloproteinase-9, *TIMP-1* tissue inhibitor of matrix metalloproteinase.

There were no differences in Lipocalin-2 serum concentrations in CLAD compared to ARAD patients (p = 0.224). However, RAS patients showed significantly higher serum concentrations of Lipocalin-2 compared to BOS (p = 0.049), ARAD (p = 0.002) and Stable-LTX patients (p = 0.009). By comparison, BOS and Stable-LTX displayed similar Lipocalin-2 serum concentrations (p = 0.868). Lipocalin-2 serum concentrations of all patient groups: LTX patients (RAS, BOS, ARAD, stable-LTX) as well as patients with end stage pulmonary fibrosis (CF and IPF) were depicted in Fig. 1A. Performing multiple testing between BOS, RAS, ARAD and stable LTX revealed no statistically significant difference in Lipocalin-2 serum concentrations (p = 0.082; Supplementary Fig. 1A).

### MMP-9 is increased in BOS compared to Stable-LTX

There were no differences in MMP-9 and TIMP-1 serum concentrations in CLAD compared to ARAD patients (p = 0.086) and (p = 0.276). However, MMP-9 serum concentrations were significantly higher in BOS compared to Stable-LTX patients (p = 0.042). TIMP-1 (p = 0.052) serum concentrations and the MMP-9/TIMP-1 ratio (p = 0.176) did not display significant differences among groups, respectively. MMP-9 and TIMP-1 serum concentrations of all patient groups: LTX patients (RAS, BOS, ARAD, stable-LTX) as well as patients with end stage pulmonary fibrosis (CF and IPF) were depicted in Fig. 1B,C. Performing multiple testing between BOS, RAS, ARAD and stable LTX revealed no statistically significant difference in MMP-9 and TIMP-1 serum concentrations (p = 0.188, p = 0.147; Supplementary Fig. 1B,C).

### Decreased Activin-A in ARAD compared to Stable-LTX and BOS

Activin-A serum concentrations were significantly decreased in ARAD patients compared to CLAD (p = 0.004), BOS (p = 0.004) and Stable-LTX (p = 0.002**)** patients. There was no difference among patients with RAS and ARAD (p = 0.305), RAS and BOS (p = 0.410), RAS and Stable-LTX (p = 0.166) or Stable-LTX and BOS patients (p = 0.601), respectively. Activin-A/Follistatin ratio was significantly decreased in ARAD compared to CLAD (p = 0.017) and BOS (p = 0.017) patients, respectively. Activin-A serum concentrations of all patient groups: LTX patients (RAS, BOS, ARAD, stable-LTX) as well as patients with end stage pulmonary fibrosis (CF and IPF) were depicted in Fig. 1D. Performing multiple testing between BOS, RAS, ARAD and stable LTX revealed significant differences in Activin-A serum concentrations (p = 0.008; Supplementary Fig. 1D).

### Low serum concentrations of Activin-A in ARAD patients

Eighty-three percent of patients with suspected BOS (41 out of 49) and 50% (6 out of 12) with suspected RAS were treated with azithromycin. While a treatment response to azithromycin was evident in 19 (46%) patients with suspected BOS, only one (16%) patient with suspected RAS was responsive to therapy. Patients with ARAD displayed reduced Activin-A serum concentrations (p = 0.001) and Activin-A/Follistatin ratio (p = 0.011) compared to patients not responding to azithromycin therapy. Activin-A serum concentrations (cut-off = 350 ng/ml) predicted responsiveness to azithromycin therapy (sensitivity = 0.772, specificity = 0.500, positive predictive value = 0.531 and negative predictive value = 0.750).

### Increased serum cytokine expression in end-stage pulmonary fibrotic diseases

**Figure 2:**
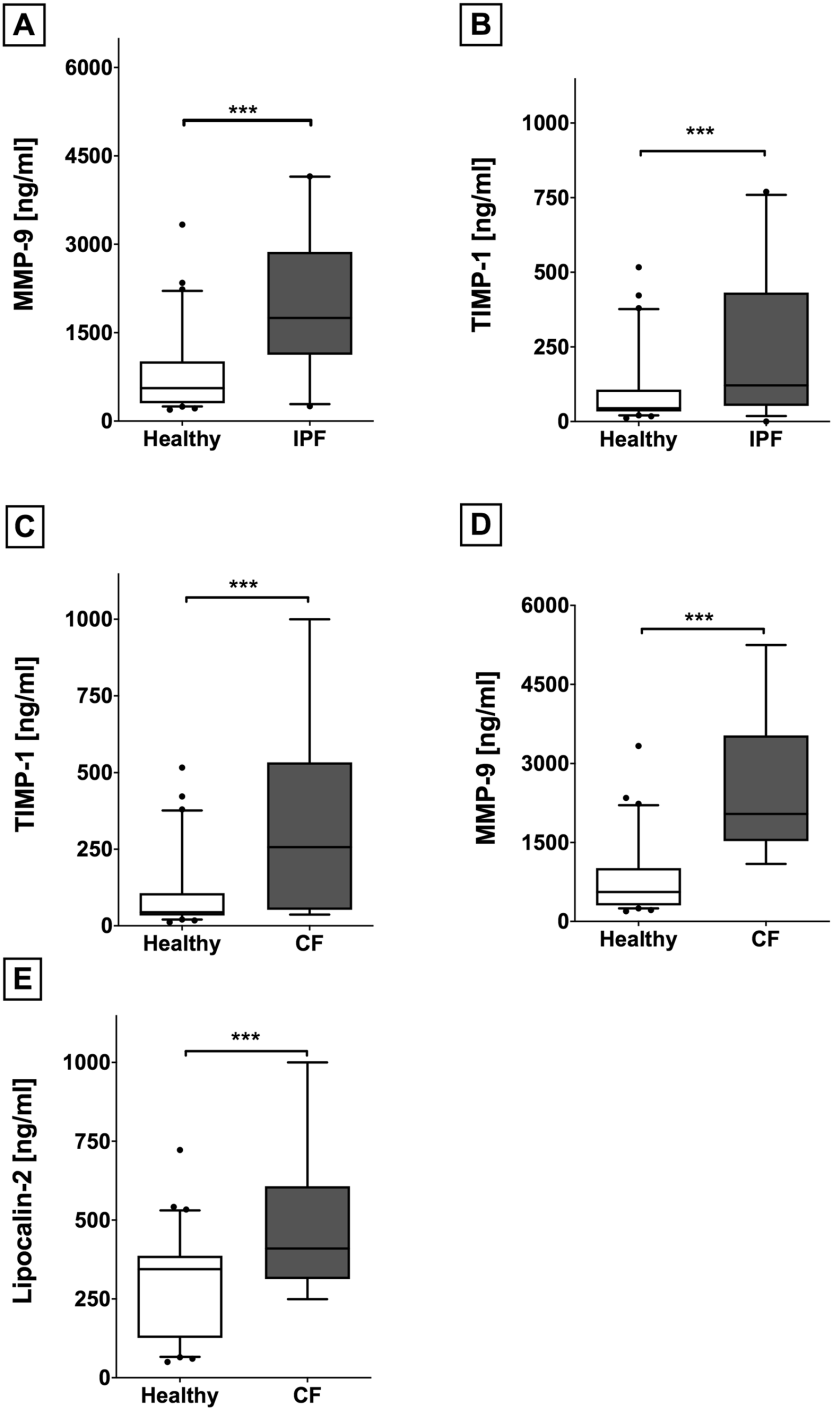
Increased expression of serum cytokines in end stage fibrotic diseases. IPF patients displayed higher serum concentrations of MMP-9 (**A**) and TIMP-1 (**B**) compared to healthy controls. CF patients also showed higher serum concentrations of MMP-9 (**C**) and TIMP-1 (**D**). Furthermore, Lipocalin-2 serum concentrations were elevated in CF patients compared to healthy volunteers (**E**). *IPF* Idiopathic pulmonary fibrosis, *MMP-9* matrix metalloproteinase-9, *TIMP-1* tissue inhibitors of metalloproteinase-1, *CF* cystic fibrosis, *RAS* restrictive allograft syndrome, *BOS* bronchiolitis obliterans syndrome.

IPF patients showed significantly elevated serum concentrations of MMP-9 (p < 0.001) and TIMP-1 (p = 0.002) compared to healthy volunteers, respectively (Fig. 2A,B). Similar to IPF, we detected higher serum concentrations of MMP-9 (p = 0.002) and TIMP-1 (p = 0.008) in CF patients (Fig. 2C,D). Furthermore, CF patients showed higher Lipocalin-2 (p = 0.007; Fig. 2E) and lower Follistatin (p = 0.024) serum concentrations than healthy controls.

### Qualitative and quantitative differences in cytokine expression between end-stage pulmonary fibrosis and CLAD

Patients with IPF had significantly lower Lipocalin-2 (p = 0.011), Follistatin (p = 0.002) and MMP-9 (p = 0.007) serum concentrations compared to CLAD patients. CLAD subtype analysis revealed significantly higher Lipocalin-2, Follistatin and MMP-9 serum concentrations in RAS (p = 0.003, p = 0.001, p = 0.049) and BOS (p = 0.029, p = 0.033, p = 0.013) compared to IPF patients, respectively. In comparison to ARAD patients, IPF patients had decreased Follistatin (p = 0.035) but increased MMP-9 (p = 0.002), Activin-A (p = 0.004) serum concentrations and Activin-A/Follistatin ratio (p = 0.035). Further, IPF patients displayed significantly decreased Follistatin (p = 0.038), however increased MMP-9 (p < 0.001) serum concentrations compared to Stable-LTX patients. Patients with CF had significantly increased MMP-9 compared to CLAD (p = 0.001), BOS (p = 0.001) and RAS (p = 0.001). Serum concentrations in CF patients were significantly lower for Follistatin (p = 0.033), but increased for Activin-A (p = 0.004), MMP-9 (p = 0.001), and Activin-A/Follistatin ratio (p = 0.001) compared to ARAD patients. Comparing CF with Stable-LTX patients MMP-9 serum concentrations (p = 0.001) and the Activin-A/Follistatin ratio (p = 0.049) were significantly increased in CF patients, respectively.

### Lipocalin-2 is expressed in bronchial epithelium and alveolar pneumocytes in RAS

**Figure 3:**
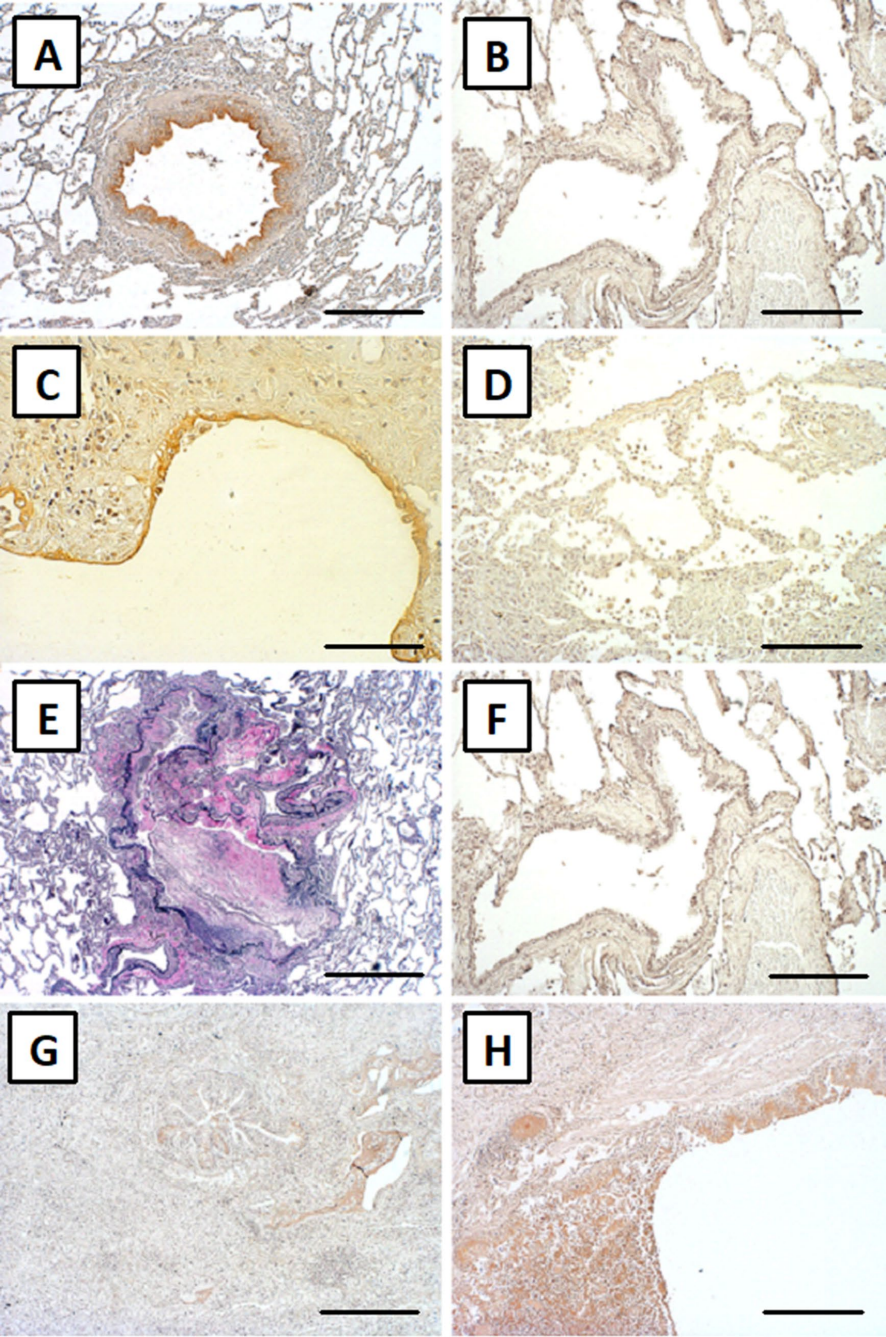
Increased pulmonary expression of Lipocalin-2 in RAS. Immunohistochemistry: Pulmonary Lipocalin-2 expression in bronchial walls of a RAS patient. [(**A**) scale bar: 400 µm]; in contrast, absent expression of Lipocalin-2 in bronchial walls of a healthy control lung [(**B**) scale bar: 400 µm]. Lipocalin-2 expression in alveolar pneumocytes of a RAS patient [(**C**) scale bar: 80 µm]. No Lipocalin-2 expression in alveolar pneumocytes of a BO patient [(**D**) scale bar: 80 µm]. Elastica-van-Gieson staining detecting BO lesions [(**E**) scale bar: 400 µm]. Adjacent slide to (**E**) showing no increased expression of Lipocalin-2 within a BO lesion [(**F**) scale bar: 400 µm]. Discrete Lipocalin-2 expression in bronchial walls of an IPF patient [(**G**) scale bar: 160 µm] and a CF patient [(**H**) scale bar: 400 µm]. *RAS* Restrictive allograft syndrome, *BO* bronchiolitis obliterans, *CF* cystic fibrosis, *IPF* idiopathic pulmonary fibrosis.

We detected Lipocalin-2 expression in the bronchial epithelium of 11 BO (100%), 6 RAS (100%), 10 IPF (100%) and 9 CF (90%) patients, but only in 5 (55.5%) healthy lung specimens by immunohistochemistry. In the lung parenchyma, only small foci of reactive alveolar pneumocyte hyperplasia showed cytoplasmic staining, which was exclusively found in 4 RAS (66.6%) and 2 IPF (20%) patients (Fig. 3 and Table 2).

**Table 2:** Semi-quantitative analysis of pulmonary Lipocalin-2 expression.

	Bronchial epithelium	Alveolar walls	Lung parenchyma	BO lesions
	Y/N (%)	Y/N (%)	Y/N (%)	Y/N (%)
BOS (n = 11)	11/0 (100)	0/11 (0)		0/11 (0)
RAS (n = 6)	6/0 (100)	4/2 (66,6)	0/6 (0)	
IPF (n = 10)	10/0 (100)	2/8 (20)	0/10(0)	
CF (n = 10)	9/1 (90)	0/10 (0)	0/10 (0)	
Control (n = 9)	5/4 (55,5)	0/9 (0)	0/9 (0)	

Immunohistochemistry for Lipocalin-2 was performed on lung specimens of patients with CLAD (RAS and BOS), end stage pulmonary fibrosis (IPF and CF) and healthy controls. Antibody reactivity in BO lesions of BOS patients, bronchial epithelium, alveolar walls and lung parenchyma was assessed.

*Y* positive antibody staining, *N* negative antibody staining, *BO* bronchiolitis obliterans, *BOS* bronchiolitis obliterans syndrome, *RAS* restrictive allograft syndrome, *IPF* idiopathic pulmonary fibrosis *CF* cystic fibrosis.

### Serum cytokines are associated with CLAD onset and survival

We investigated the correlation of cytokine serum concentrations and clinical outcome, including future onset of CLAD and OS or re-transplantation. The median time between serum sampling and onset of CLAD was at 62.2 (range 84.7) months and for overall survival or re-transplantation at 62.3 (range 85.2) months. Eight Stable-LTX patients (12.7%) developed CLAD during follow-up. Of those patients, three underwent extracorporeal photopheresis (37.5%) for 5.8–30.0 months (mean = 14.6 months). Of 63 Stable-LTX patients, 11 died during follow up (17.4%). Among them, four died from later developed CLAD (36.4%; 3 BOS and 1 RAS), five died from infections (45.5%), one from cerebral bleeding (9.1%) and one patient died from unknown causes (9.1%).

**Figure 4:**
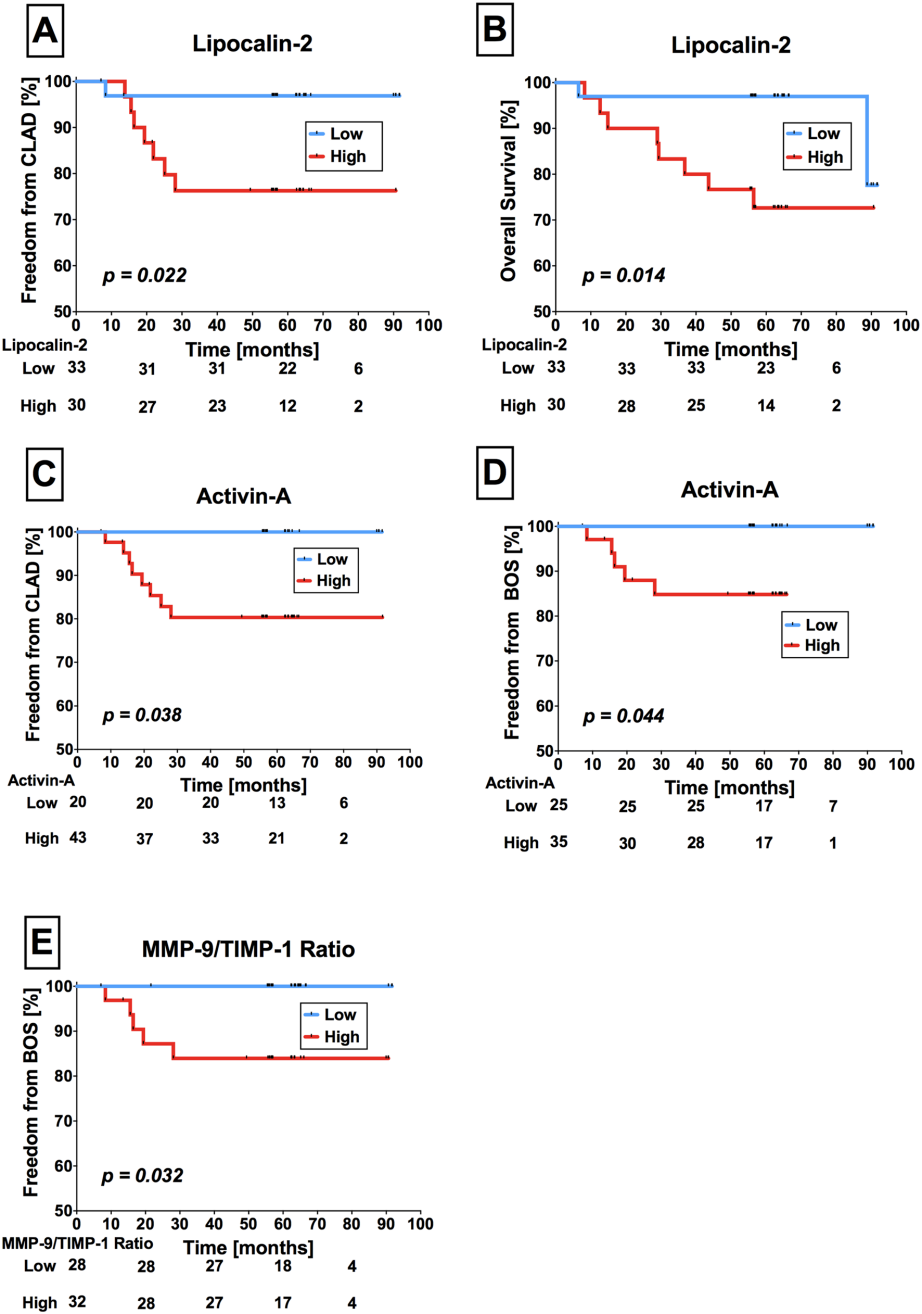
Lipocalin-2 serum concentrations in Stable-LTX predict future onset of CLAD and BOS. Stable-LTX patients with high Lipocalin-2 serum concentrations were significantly more likely to develop CLAD during follow-up compared to those with low Lipocalin-2 concentrations (**A**). Higher Lipocalin-2 serum concentrations in Stable-LTX patients resulted in worse OS during follow-up (**B**). High Activin-A serum concentrations were also associated with higher CLAD onset rates in Stable-LTX patients (**C**). Higher serum concentrations of Activin-A where significantly associated with the onset of CLAD subtype BOS (**D**). High MMP-9/TIMP-1 ratios translated to higher BOS onset rates compared to low MMP-9/TIMP-1 ratios in Stable-LTX patients (**E**). *LTX* Lung transplantation, *CLAD* chronic lung allograft dysfunction, *RAS* restrictive allograft syndrome, *BOS* bronchiolitis obliterans syndrome, *MMP-9* matrix metalloproteinase-9, *TIMP-1* tissue inhibitors of metalloproteinase-1.

High serum concentrations of Lipocalin-2 were significantly associated with earlier onset of CLAD (Cut-off = 350.4 ng/ml, sensitivity = 0.875, specificity = 0.418, p = 0.022) and adverse OS (Cut-off = 354.1 ng/ml, sensitivity = 0.800, specificity = 0.604, p = 0.014) (Fig. 4A,B). Higher Activin-A serum concentrations were significantly associated with onset of CLAD (Cut-off = 217 ng/ml, sensitivity = 1.000, specificity = 0.364, p = 0.038) (Fig. 4C). With regard to CLAD phenotypes, higher Activin-A serum concentrations (Cut-off = 347 ng/ml, sensitivity = 0.800, specificity = 0.564, p = 0.044) (Fig. 4D) and higher MMP-9/TIMP-1 ratios (Cut-off = 4.1, sensitivity = 1.000, specificity = 0.509, p = 0.032) (Fig. 4E) predicted onset BOS, but not RAS patients.

## Discussion

While milestones in the perioperative management of LTX were achieved, CLAD, manifesting as BOS or RAS remains the main reason for adverse long-term outcome. The underlying pathomechanisms of the CLAD phenotypes are barely understood and an accurate diagnosis is often difficult to obtain. Biomarkers aiding in diagnosis and identifying pathophysiological pathways might spark new developments in CLAD management.

Our data identified Lipocalin-2 as a potentially useful biomarker to distinguish RAS from BOS and Stable-LTX. Lipocalin-2 is constitutively expressed and stored in neutrophilic granules making it a possible biomarker for neutrophil activity^17^. However, previous studies did not show differences in neutrophil counts in lungs of RAS compared to BOS patients^18^. Thus, whether elevated levels of circulating Lipocalin-2 in RAS patients stem from increased neutrophil activity remains elusive. A possible source of serum Lipocalin-2 might be secreted Lipocalin-2 expressed in the bronchial epithelium of all BOS and RAS patients (as detected by our immunohistochemistry).

In the case of increased serum Lipocalin-2 in end-stage CF, neutrophils are a plausible source. The airways of adult CF patients are chronically besieged by neutrophilic inflammation and circulating Lipocalin-2 was reported to be elevated in acute exacerbations and Pseudomonas aeruginosa infections of CF patients^19^. It is strongly and selectively induced in bronchial and alveolar cells of inflamed lungs by Interleukin-1β^20^. We found Lipocalin-2 expression in epithelial cells of 90% of all CF patients. In line with our findings in IPF are correlations of Lipocalin-2 BAL levels with BAL neutrophilia and forced vital capacity, as well as no elevation of plasma Lipocalin-2. Further, Lipocalin-2 was expressed in airway epithelial cells that covered the honeycomb cysts and in bronchioles of IPF patients^21^.

Lipocalin-2 is known to build complexes with MMP-9, thereby stabilizing MMP-9 activity^22^. Airway remodeling is promoted by active secretion of MMP-9 by bronchial epithelial cells triggered by T-cells^23^ In accordance to these findings, several studies linked elevated MMP-9 concentrations and MMP-9/TIMP-1 ratios in serum and BAL to CLAD, IPF and CF^24,25^. We detected higher MMP-9 serum concentrations (but not MMP-9/TIMP-1 ratio) in BOS patients compared to Stable-LTX patients and lower MMP-9/TIMP-1 ratios in CLAD compared to CF and IPF patients, suggesting a different importance of protease-antiprotease imbalance in pathogenesis. Divergent study results concerning cytokine profiles may pertain to different immunosuppressive therapy regimens. In previous studies, TIMP-1 was shown to be up-regulated in human pulmonary fibrosis^26^. Our study supported these results since TIMP-1 serum concentrations were higher in end-stage CF and IPF compared to healthy volunteers, but not compared to Stable-LTX or CLAD patients*.*

In a study on patients after LTX Activin-A serum concentrations decreased from postoperative week two to week twelve, while Follistatin serum concentrations remained unchanged until they increased 24 weeks post-LTX. Patients with primary graft dysfunction expressed lower serum Follistatin and higher Activin-A/follistatin ratios^27^. Our study was neither performed in the perioperative setting nor during a dramatic event such as primary graft dysfunction. We discovered Activin-A as a promising biomarker to distinguish ARAD patients from Stable-LTX and BOS patients, however not from RAS. Activin-A served as a predictive molecule to measure the responsiveness to azithromycin therapy. Low Activin-A concentrations in ARAD patients are in line with previous studies showing that Activin-A promotes inflammation at lower concentrations mediated by resting monocytes/macrophages, but inhibits inflammatory activity as concentrations increase^16^. Azithromycin was shown to exhibit inhibitory effects on neutrophils, such as diminishing neutrophil extracellular traps release^28^. Activin-A serum concentrations were not elevated in IPF and CF patients. Interestingly, Activin-A serum concentrations were reported to correlate inversely with lung function and nutritional status in CF patients^29^. In a CF mouse model with CF-like lung disease, Follistatin administration reduced Activin-A levels, mucus hypersecretion, and airway neutrophilia. ln CF patients, decreased serum concentrations of Follistatin and elevated Activin-A resulted in an increased Activin-A/Follistatin ratio^29^. While we detected decreased serum concentrations of Follistatin, we did not observe elevated Activin-A concentrations and Activin-A/Follistatin ratios in end-stage CF patients. These different results might stem from different CF disease severity: the aforementioned study sampled blood from stable CF patients during outpatient visits, while our study investigated patients with end-stage pulmonary CF immediately before LTX. In our study, neither Activin-A serum concentrations nor Activin-A/Follistatin ratios were elevated in end-stage IPF patients.

Our study has several limitations due to the retrospective analysis of prospectively collected blood samples, the single center experience and the limited sample size. Large-scale studies are warranted for further biomarker evaluation in CLAD, ARAD, CF and IPF patients. We are not suggesting that our absolute serum concentration values can be used to make any judgments about patient’s diagnosis. While comparative results during one experiment could be repeated in separate ELISA tests. The commercially available ELISA tests were only sold for research use and not for diagnostic purposes. Since cut-offs were chosen via Youden Index out of our study results, sensitivity and specificity must be interpreted with caution. Early recognition of different CLAD phenotypes is essential for developing new therapies and improving outcomes. This study identified promising new biomarkers for better characterization and classification of CLAD phenotypes in addition to well-established diagnostic methods including spirometry and radiological imaging. Lipocalin-2 might serve as a potential diagnostic or even therapeutic target for RAS patients. Activin-A seems helpful to identify ARAD patients. Further prospective studies will be required confirming our biomarker results. The statistically significant difference in Lipocalin-2 serum concentrations between BOS and RAS deserves further investigations; as does Activin-A in ARAD patients by employing further methods including immunostaining.

## Materials and methods

**Figure 5:**
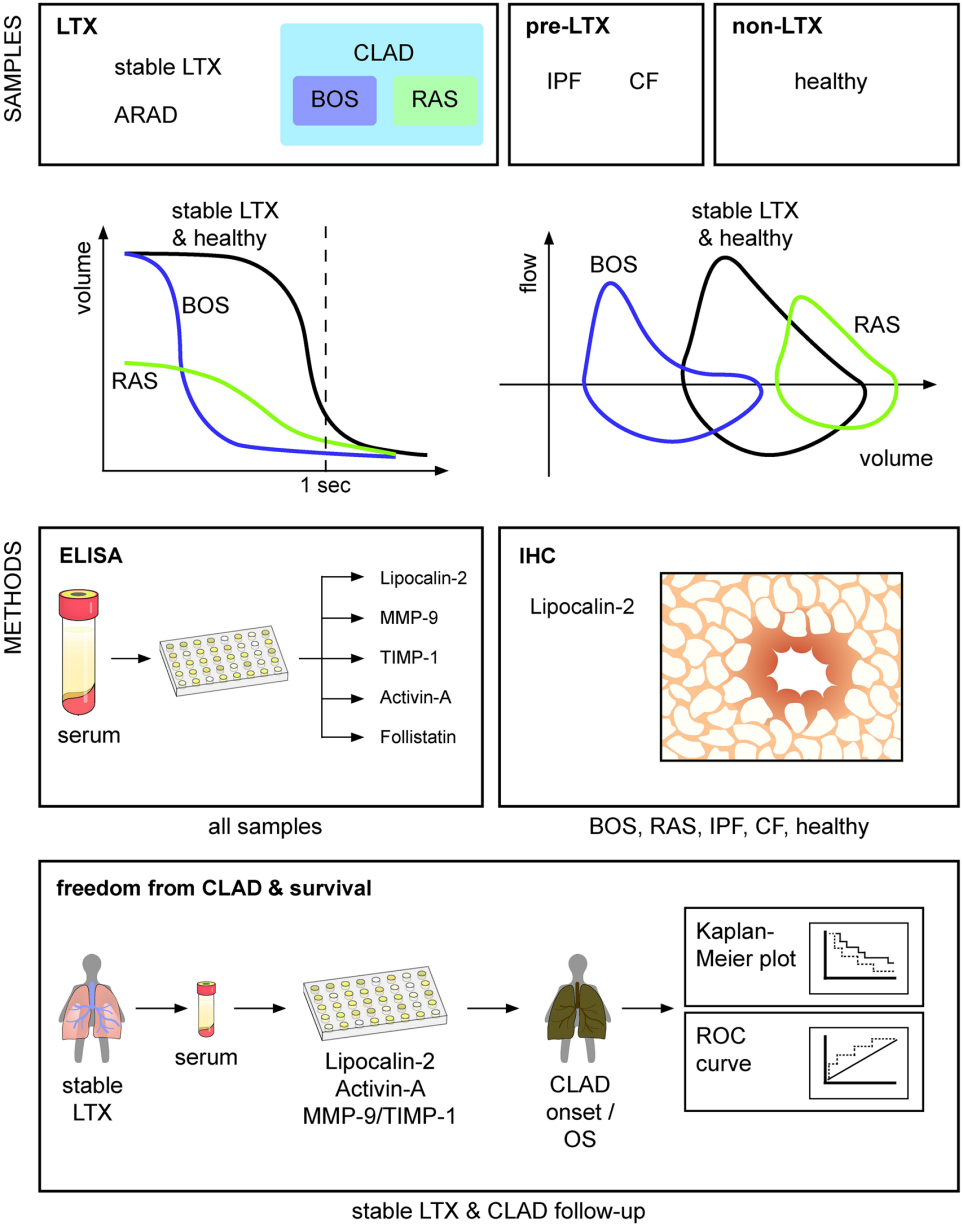
Graphical depiction of the study design. Serum samples were selected of LTX patients diagnosed with CLAD (BOS (n = 30) and RAS (n = 11)), ARAD (n = 22) and Stable-LTX (n = 56), patients with end-stage pulmonary disease [IPF (n = 31) and CF (n = 15)] listed for LTX and healthy volunteers (n = 63). CLAD phenotypes, Stable-LTX and ARAD patients were diagnosed via spirometry. Prototypical spirometric volume changes over time and flow volume loops of stable lung transplanted patients/healthy people and patients with BOS and RAS are depicted. Enzyme-linked immunosorbent assays were employed for measuring cytokine serum concentrations of Lipocalin-2, MMP-9, TIMP-1, Activin-A and Follistatin. MMP-9/TIMP-1 and Activin-A/Follistatin ratios were calculated. Immunohistochemical stainings were performed in specimens of 20 patients who underwent re-transplantation for either BOS (n = 11) or RAS (n = 9), 20 patients who underwent primary LTX for either IPF (n = 10) or CF (n = 10), and 10 patients who served as healthy controls. Further, the correlation of cytokine serum concentrations and clinical outcome, including OS, future onset of CLAD and re-transplantation was analysed by performing Kaplan–Meier analysis. *LTX* Lung transplantation, *CLAD* chronic lung allograft dysfunction, *BOS* bronchiolitis obliterans syndrome, *RAS* restrictive allograft syndrome, *ARAD* azithromycin-reversible allograft dysfunction, *IPF* idiopathic pulmonary fibrosis, *CF* cystic fibrosis, *MMP-9* matrix metalloproteinase-9, *TIMP-1* tissue inhibitors of metalloproteinase-1.

This single-centre explorative cohort study was performed at the institutional Division of Thoracic Surgery. All methods were carried out in accordance with relevant guidelines and regulations in the manuscript. Ethical approval was obtained from the institutional Ethics Committee of the medical university of vienna (EC-No:846/2010). All patients provided written informed consent prior to participation in the study. A graphical depiction of the study design is shown in Fig. 5.

### Definition of CLAD phenotypes and ARAD

CLAD was defined as persistent pulmonary function decline (FEV_1_ ≥ 20%) calculated from baseline (the best two FEV_1_ measurements after LTX performed more than three weeks apart) after exclusion of non-CLAD causes by pulmonary function testing, bronchoscopy including transbronchial/endobronchial biopsies and BAL, blood work and radiological imaging. Patients suffering from suspected CLAD were treated with azithromycin 250–500 mg three times per week (irrespective of BAL neutrophilia) for 3 months. Patients with an FEV_1_ improvement greater than 10% were classified as ARAD and not included in the analysis of CLAD. CLAD was further subdivided into RAS (concomitant drop in Total Lung Capacitiy (TLC) ≥ 10% compared to baseline levels and persistent opacities on chest imaging) and BOS (no restrictive pulmonary function pattern)^6^.

### Patient cohort

Demographic and clinical data were depicted in Table 3. For serum analysis, we enrolled 119 patients undergoing LTX. Among them, 41 patients were diagnosed with CLAD and dichotomized into BOS (n = 30) and RAS (n = 11) phenotypes. The included BOS patients displayed the following disease severity: BOS1 (n = 5), BOS2 (n = 13) and BOS3 (n = 12). Ten out of 11 RAS patients had a mixed phenotype.

**Table 3:** Demographic and clinical data.

	Stable-LTX (n = 63)	ARAD (n = 22)	CLAD (n = 41)	BOS (n = 30)	RAS (n = 11)	IPF (n = 23)	CF (n = 21)
Age mean ± SD	50.6 ± 14.3	50.4 ± 14.3	51.0 ± 13.5	49.8 ± 13.2	55.9 ± 14.0	51.7 ± 11.1	31.0 ± 11.7
Gender F/M ratio	0.966	0.953	0.906	1.042	0.500	0.381	1.286
**Diagnosis prior to TX**							
COPD n *(%)*	37 *(58.7)*	9 *(40.9)*	18 *(43.9)*	14 *(46.6)*	*6 (54.5)*	–	–
IPF n *(%)*	8 *(12.6)*	7 *(31.8)*	8 *(19.5)*	*6 (20.0)*	*2 (18.8)*	23 *(100.0)*	21 *(100.0)*
IPAH n *(%)*	4 *(6.3)*	1 *(4.5)*	4 *(9.7)*	3 *(10.0)*	*1 (9.0)*	–	–
CF n *(%)*	8 *(12.6)*	5 *(22.7)*	4 *(9.7)*	4 *(13.3)*	–	–	–
Others n *(%)*	5 *(7.9)*	–	5 *(12.1)*	3 *(10.0)*	2 *(18.8)*	–	–
**Type of TX**							
DLUTX n *(%)*	52 *(82.5)*	20 *(90)*	33 *(80.4)*	24 *(80.0)*	9 *(81.8)*	–	–
SLUTX n *(%)*	10 *(15)*	2 *(9.0)*	7 *(17.0)*	5 *(16.6)*	2 *(18.1)*	–	–
HLTX n *(%)*	1 *(1.5)*	0 *(0.0)*	1 *(2.4)*	1 *(3.3)*	–	–	–
**CMV risk**							
D+/R− n *(%)*	15 *(23.8)*	5 *(22.7)*	9 *(21.1)*	8 *(26.6)*	*1 (9.0)*	–	–
D+/R+ n *(%)*	30 *(47.6)*	10 *(45.5)*	19 *(46.3)*	15 *(50.0)*	4 *(36.3)*	–	–
D−/R+ n *(%)*	12 *(19.0)*	4 *(18.1)*	7 *(17.0)*	4 *(13.3)*	3 *(27.2)*	–	–
D−/R− n *(%)*	5 *(1.5)*	3 *(15.0)*	6 *(14.6)*	3 *(10.0)*	3 *(27.2)*	–	–

*Stable LTX* Lung transplantation, *ARAD* sa*, CLAD* chronic lung allograft dysfunction, *BOS* bronchiolitis obliterans syndrome, *RAS* restrictive allograft syndrome, *IPF* idiopathic pulmonary fibrosis, *CF* cystic fibrosis, *n* number of individuals, *SD* standard deviation;

Patients with ARAD (n = 22) were separately analysed and not included in the CLAD cohort. Sixty-three LTX patients with stable pulmonary function were included in this study (Stable-LTX). Blood samples of patients with stable-LTX were drawn at clinical routine check-up including surveillance bronchoscopy. Furthermore we enrolled patients with end-stage IPF (n = 31) and CF (n = 15) listed for LTX. Serum samples of 63 healthy volunteers served as a control group. None of the healthy volunteers suffered from acute or chronic diseases, such as asthma, chronic obstructive pulmonary disease, acute or chronic kidney diseases, autoimmune diseases or any cancer type. Furthermore, none of the healthy volunteers took medical drugs for at least three months before the blood draw.

Blood samples were aliquoted after centrifugation with 2851×*g* for 15 min at 4 °C and stored in 2 ml cryotubes at − 80 °C in until further analysis.

CLAD, Stable-LTX, ARAD and control groups were matched by gender and age. There were no statistically significant gender differences in any of the study groups (CLAD, comprising BOS and RAS, ARAD, stable-LTX and healthy volunteers). Mean age of the study groups ranged from 49.8 years in BOS to 51.7 years in IPF patients. There were no statistically significant age differences between ARAD, BOS, RAS, Stable-LTX and IPF patients. However, CF patients were significantly younger (mean age of 31.0 years) compared to patients with ARAD, BOS, RAS, stable LTX and IPF.

### Standard immunosuppressive treatments

Fifty patients (42.0%) received induction therapy with either alemtuzumab (n = 34, 68.0%), anti-thymocyte globulin (n = 15, 30.0%) or daclizumab (n = 1, 2.0%). Maintenance therapy was performed in all patients with tacrolimus, mycophenolat-mofetil (only in the non-alemtuzumab group) and corticosteroids. All patients undergoing LTX received postoperative anti-infectious prophylaxis therapy with piperazillin/tazobactam, a lifelong pneumocystis prophylaxis with trimethoprim-sulfamethoxazole, prophylactic inhalation therapy with amphotericin B and gentamicin; and cytomegalovirus (CMV) prophylaxis including CMV hyperimmunoglobulines, together with valganciclovir.

### Measurement of cytokine serum concentrations

Enzyme-Linked Immunosorbent Assays (ELISA) were employed for measuring cytokine serum concentrations according to the manufacturer’s protocol, using commercially available DuoSet ELISA kits: Human Lipocalin-2/NGAL, Human MMP-9, Human TIMP-1, Human Activin-A, Human Follistatin (R&D Systems, Minneapolis, MN 55413, USA). Accordingly, MMP-9/TIMP-1 and Activin-A/Follistatin ratios were calculated. Serum samples were diluted with reagent diluent for the measurements of Lipocalin-2 and TIMP-1 (500-fold), MMP-9 (5000-fold), and Follistatin (twofold) but not Activin-A (no dilution).

### Immunohistochemistry

Immunohistochemistry was performed with specimens of 50 patients allocated to the following subgroups: 20 patients who underwent re-transplantation for either BOS (n = 11) or RAS (n = 9), 20 patients who underwent primary LTX for either IPF (n = 10) or CF (n = 10) and 10 patients who served as healthy controls. Patients of the latter group underwent lobectomy for peripherally located lung cancer (pT1pN0M0). The tumour was located at least five centimetres from the investigated lung area and patients showed no evidence for advanced parenchymal lung diseases.

Immunohistochemical staining was performed on formaldehyde-fixed, paraffin-embedded tissue specimens according to routine protocols^30^. Anti-Lipocalin-2/NGAL antibody ab115324 (Abcam, Cambridge, UK) and anti-goat IgG secondary antibodies (Vector Laboratories, Burlingame, CA, USA) were used for staining. Omission of the primary antibody served for negative control staining. Results were assessed by two independent investigators, including one specialist on pulmonary histopathology (O.F.). Antibody staining of alveolar lining cells, bronchial epithelium and the interstitium were evaluated on each slide. In case of interobserver discrepancies, results were discussed until consensus was achieved. BOS lesions were identified by Elastica van Gieson staining on adjacent slides.

### Statistical methods

Statistical data analysis was performed using SPSS software (Version 20; IBM SPSS Inc., Chicago, IL, USA). Graphical methods (histograms) were employed to test normality. Data were reported as median (range) for non-normal distributions. Mann–Whitney U test was used to compare two independent groups. Kruskal–Wallis rank test was used to perform multiple testing followed by Dunn’s multiple comparison tests. *P*-values < 0.05 were considered statistically significant**.** For follow-up analysis, cytokine serum concentrations were dichotomized into low and high subgroups by using *ROC*-Curves for calculating Youden-Indices. We used the best cut-offs for maximizing sensitivity at the expense of specificity in order to create a test that is maximally sensitive. Accordingly, we performed Kaplan–Meier survival analysis and used log-rank analysis for outcome assessment. For the analysis of the prognostic value of serum cytokines, the date of serum withdrawal was taken as the starting point for follow-up analysis. We analysed Stable-LTX patients for freedom from CLAD and OS. In order to express the precision and repeatability of our ELISA experiments we calculated the intra-assay and inter-assay CV. Therefore, we performed following calculation % CV = SD of plate means ÷ mean of plate means × 100. GraphPad Prism 5 (GraphPad Software, La Jolla, CA, USA) was used for data visualization. Boxplots were designed as follows: box: 1st to 3rd quartile, bar: median, whiskers: percentile 5–95, outliers: all shown as dots.

## Supplementary Information

Supplementary Information.

## Data Availability

All data generated or analysed during this study are included in this published article.

## Abbreviations

ARAD: Azithromycin-responsive allograft dysfunction
BAL: Bronchioalveolar lavage
BOS: Bronchiolitis obliterans syndrome
BO: Bronchiolitis obliterans
CLAD: Chronic lung allograft dysfunction
CF: Cystic fibrosis
ELISA: Enzyme-linked immunosorbent assay
FEV1: Forced expiratory volume in one second
IPF: Idiopathic pulmonary fibrosis
LTX: Lung transplantation
MMP-9: Matrix metalloproteinase-9
OS: Overall survival
RAS: Restrictive allograft syndrome
TIMP-1: Tissue inhibitors of metalloproteinase-1
TLC: Total lung capacity

## References

[1] Verleden GM, Raghu G, Meyer KC, Glanville AR, Corris P (2014). A new classification system for chronic lung allograft dysfunction. J. Heart Lung Transplant..

[2] Meyer KC, Raghu G, Verleden GM (2014). An international ISHLT/ATS/ERS clinical practice guideline: Diagnosis and management of bronchiolitis obliterans syndrome. Eur. Respir. J..

[3] Nishikawa T, Inomata S, Igarashi M, Goyagi T, Naito H (1992). Plasma lidocaine concentrations during epidural blockade with isoflurane or halothane anesthesia. Anesth. Analg..

[4] Sato M, Waddell TK, Wagnetz U (2011). Restrictive allograft syndrome (RAS): A novel form of chronic lung allograft dysfunction. J. Heart Lung Transplant..

[5] Sato M, Hwang DM, Waddell TK, Singer LG, Keshavjee S (2013). Progression pattern of restrictive allograft syndrome after lung transplantation. J. Heart Lung Transplant..

[6] Verleden GM, Vos R, Vanaudenaerde B (2015). Current views on chronic rejection after lung transplantation. Transpl. Int..

[7] Fernandez IE, Eickelberg O (2012). New cellular and molecular mechanisms of lung injury and fibrosis in idiopathic pulmonary fibrosis. Lancet.

[8] Walshaw MJ (2019). Cystic fibrosis: Diagnosis and management—NICE guideline 78. Paediatr. Respir. Rev..

[9] Castellani S, Di Gioia S, di Toma L, Conese M (2018). Human cellular models for the investigation of lung inflammation and mucus production in cystic fibrosis. Anal. Cell Pathol. (Amst)..

[10] Fernandez IE, Heinzelmann K, Verleden S, Eickelberg O (2015). Characteristic patterns in the fibrotic lung. Comparing idiopathic pulmonary fibrosis with chronic lung allograft dysfunction. Ann. Am. Thorac. Soc..

[11] Dittrich AM, Meyer HA, Hamelmann E (2013). The role of lipocalins in airway disease. Clin. Exp. Allergy..

[12] Redl B, Wojnar P, Ellemunter H, Feichtinger H (1998). Identification of a lipocalin in mucosal glands of the human tracheobronchial tree and its enhanced secretion in cystic fibrosis. Lab. Investig..

[13] Flo TH, Smith KD, Sato S (2004). Lipocalin 2 mediates an innate immune response to bacterial infection by sequestrating iron. Nature.

[14] Gueders MM, Foidart JM, Noel A, Cataldo DD (2006). Matrix metalloproteinases (MMPs) and tissue inhibitors of MMPs in the respiratory tract: Potential implications in asthma and other lung diseases. Eur. J. Pharmacol..

[15] Karagiannidis C, Hense G, Martin C (2006). Activin A is an acute allergen-responsive cytokine and provides a link to TGF-beta-mediated airway remodeling in asthma. J. Allergy Clin. Immunol..

[16] Hedger MP, de Kretser DM (2013). The activins and their binding protein, follistatin-Diagnostic and therapeutic targets in inflammatory disease and fibrosis. Cytokine Growth Factor Rev..

[17] Cruz DN, Gaiao S, Maisel A, Ronco C, Devarajan P (2012). Neutrophil gelatinase-associated lipocalin as a biomarker of cardiovascular disease: A systematic review. Clin. Chem. Lab. Med..

[18] Vandermeulen E, Lammertyn E, Verleden SE (2017). Immunological diversity in phenotypes of chronic lung allograft dysfunction: A comprehensive immunohistochemical analysis. Transpl. Int..

[19] Zughaier SM, Tangpricha V, Leong T, Stecenko AA, McCarty NA (2013). Peripheral monocytes derived from patients with cystic fibrosis and healthy donors secrete NGAL in response to *Pseudomonas aeruginosa* infection. J. Investig. Med..

[20] Cowland JB, Sorensen OE, Sehested M, Borregaard N (2003). Neutrophil gelatinase-associated lipocalin is up-regulated in human epithelial cells by IL-1 beta, but not by TNF-alpha. J. Immunol..

[21] Ikezoe K, Handa T, Mori K (2014). Neutrophil gelatinase-associated lipocalin in idiopathic pulmonary fibrosis. Eur. Respir. J..

[22] Bouchet S, Bauvois B (2014). Neutrophil gelatinase-associated lipocalin (NGAL), pro-matrix metalloproteinase-9 (pro-MMP-9) and their complex pro-MMP-9/NGAL in leukaemias. Cancers (Basel)..

[23] Pain M, Royer PJ, Loy J (2017). T cells promote bronchial epithelial cell secretion of matrix metalloproteinase-9 via a C-C chemokine receptor type 2 pathway: Implications for chronic lung allograft dysfunction. Am. J. Transplant..

[24] Kennedy VE, Todd JL, Palmer SM (2013). Bronchoalveolar lavage as a tool to predict, diagnose and understand bronchiolitis obliterans syndrome. Am. J. Transplant..

[25] Heijink IH, Rozeveld D, van der Heide S (2015). Metalloproteinase profiling in lung transplant recipients with good outcome and bronchiolitis obliterans syndrome. Transplantation.

[26] Lagente V, Boichot E (2010). Role of matrix metalloproteinases in the inflammatory process of respiratory diseases. J. Mol. Cell Cardiol..

[27] Westall GP, Snell GI, Loskot M (2017). Activin biology after lung transplantation. Transplant Direct..

[28] Bystrzycka W, Manda-Handzlik A, Sieczkowska S, Moskalik A, Demkow U, Ciepiela O (2017). Azithromycin and chloramphenicol diminish neutrophil extracellular traps (NETs) release. Int. J. Mol. Sci..

[29] Hardy CL, King SJ, Mifsud NA (2015). The activin A antagonist follistatin inhibits cystic fibrosis-like lung inflammation and pathology. Immunol. Cell Biol..

[30] Janik S, Schiefer AI, Bekos C (2016). HSP27 and 70 expression in thymic epithelial tumors and benign thymic alterations: Diagnostic, prognostic and physiologic implications. Sci. Rep..

