# Supplementary material for: Potential novel biomarkers for chronic lung allograft dysfunction and azithromycin responsive allograft dysfunction
Source: Sci Rep. 2021 Mar 24;11:6799. doi: 10.1038/s41598-021-85949-1 (PMC7990920; doi:10.1038/s41598-021-85949-1)
Supplement: Supplementary file 1 — Supplementary Information. [file 41598_2021_85949_MOESM1_ESM.docx]

**Potential novel biomarkers for chronic lung allograft dysfunction and azithromycin responsive allograft dysfunction**

Cecilia Veraar, MD^1*^, Jonathan Kliman, MD^2*^, Alberto Benazzo, MD^2^, Felicitas Oberndorfer, MD^3^, Maria Laggner, PhD^2^, Philipp Hacker, MD^2^, Thomas Raunegger, MD^2^, Stefan Janik, PhD^4^, Peter Jaksch, MD^2^, Walter Klepetko, MD^2^, Hendrik J Ankersmit, MD^2,5,6^, Bernhard Moser, MD, MBA^2^

^1^ Department of Anaesthesiology, General Intensive Care and Pain Medicine, Division of Cardiac Thoracic Vascular Anaesthesia and Intensive Care Medicine, Medical University of Vienna, Vienna, Austria.

^2^ Department of Thoracic Surgery, Division of Surgery, Medical University of Vienna, Vienna, Austria.

^3^ Clinical Institute of Pathology, Medical University of Vienna, Vienna, Austria.

^4^ Department of Otorhinolaryngology, Head and Neck Surgery, Medical University of Vienna, Vienna, Austria.

^5^ Christian Doppler Laboratory for Diagnosis and Regeneration of Cardiac and Thoracic Diseases, Medical University of Vienna, Vienna, Austria.

^6^ Head FFG Project "APOSEC", FOLAB Surgery, Medical University of Vienna, Vienna, Austria.

^*^contributed equally to the manuscript

**Corresponding Author**

Bernhard Moser, MD, PD, Assoc. Prof., MBA, FEBTS. Department of Surgery, Division of Thoracic Surgery, Medical University of Vienna. Waehringer Guertel 18-20. 1090 Vienna, Austria. E-mail: bernhard.moser@meduniwien.ac.at; Phone +43 1 40400 67770.

**Supplementary Table 1: Inter-and intra-assay coefficients of variation for ELISA experiments**

| **ELISA** | **LCN-2** | **MMP-9** | **TIMP-1** | **Activin-A** | **FST** |
| --- | --- | --- | --- | --- | --- |
| intra-assay mean of %CV | **1.0** | **1.8** | **3.2** | **2.3** | **3.4** |
| inter-assay %CV | **6.3** | **7.4** | **11.2** | **9.7** | **9.5** |

*LCN-2* Lipocalin-2, *MMP-9* Matrix Metalloproteinase-9, *TIMP-1* Tissue Inhibitor of Matrix Metalloproteinase, *FST* Follistatin, *%CV* coefficient of variation

**Supplementary Figure 1: Multiple testing of ARAD, stable-LTX, RAS and BOS patients according to biomarkers**

There were no statistically significant (**A**) Lipcalin-2, (**B**) MMP-9 and (**C**) TIMP-1 serum concentrations between ARAD, stable-LTX, BOS and RAS patients. (**D**) Activin-A serum concentrations revealed statistically significant differences between ARAD, stable-LTX, BOS and RAS patients. Kruskal-Wallis rank test was used to perform multiple testing followed by Dunn’s multiple comparison tests.

*P < 0.05; **P < 0.01; ***P < 0.001

*RAS* Restrictive Allograft Syndrome, *BOS* Bronchiolitis Obliterans Syndrome, *ARAD* Azithromycin-Responsive Allograft Dysfunction, *MMP-9* Matrix Metalloproteinase-9, *TIMP-1* Tissue Inhibitor of Matrix Metalloproteinase
